# In-Situ Screening of Soybean Quality with a Novel Handheld Near-Infrared Sensor

**DOI:** 10.3390/s20216283

**Published:** 2020-11-04

**Authors:** Didem Peren Aykas, Christopher Ball, Amanda Sia, Kuanrong Zhu, Mei-Ling Shotts, Anna Schmenk, Luis Rodriguez-Saona

**Affiliations:** 1 Department of Food Science and Technology, The Ohio State University, 100 Parker Food Science and Technology Building, 2015 Fyffe Road, Columbus, OH 43210, USA; aykas.1@osu.edu (D.P.A.); sia.6@buckeyemail.osu.edu (A.S.); zhu.1421@buckeyemail.osu.edu (K.Z.); mei.shotts@abbott.com (M.-L.S.); schmenk.32@buckeyemail.osu.edu (A.S.); 2 Department of Food Engineering, Faculty of Engineering, Adnan Menderes University, Aydin 09100, Turkey; 3 ElectroScience Laboratory, The Ohio State University, 1330 Kinnear Road, Columbus, OH 43212, USA; ball.51@osu.edu

**Keywords:** soybean, protein content, essential amino acids, fat content, major fatty acids, near-infrared spectroscopy, partial least square regression, SIMCA

## Abstract

This study evaluates a novel handheld sensor technology coupled with pattern recognition to provide real-time screening of several soybean traits for breeders and farmers, namely protein and fat quality. We developed predictive regression models that can quantify soybean quality traits based on near-infrared (NIR) spectra acquired by a handheld instrument. This system has been utilized to measure crude protein, essential amino acids (lysine, threonine, methionine, tryptophan, and cysteine) composition, total fat, the profile of major fatty acids, and moisture content in soybeans (*n* = 107), and soy products including soy isolates, soy concentrates, and soy supplement drink powders (*n* = 15). Reference quantification of crude protein content used the Dumas combustion method (AOAC 992.23), and individual amino acids were determined using traditional protein hydrolysis (AOAC 982.30). Fat and moisture content were determined by Soxhlet (AOAC 945.16) and Karl Fischer methods, respectively, and fatty acid composition via gas chromatography-fatty acid methyl esterification. Predictive models were built and validated using ground soybean and soy products. Robust partial least square regression (PLSR) models predicted all measured quality parameters with high integrity of fit (R_Pre_ ≥ 0.92), low root mean square error of prediction (0.02–3.07%), and high predictive performance (RPD range 2.4–8.8, RER range 7.5–29.2). Our study demonstrated that a handheld NIR sensor can supplant expensive laboratory testing that can take weeks to produce results and provide soybean breeders and growers with a rapid, accurate, and non-destructive tool that can be used in the field for real-time analysis of soybeans to facilitate faster decision-making.

## 1. Introduction

Soybeans (*Glycine max* (L.) Merr.) are one of the most valuable legume crops worldwide, with global production reaching 361 million metric tons in 2019 [1]. The soybean’s economic viability comes from both its high protein (~38%) and oil (~18%) content [2]. In addition to providing up to 90% of the U.S. oilseed production; soybeans are the largest source of animal protein feed globally [3]. High protein content combined with an excellent profile of essential amino acids that are highly digestible makes the soybean a valuable source of protein for livestock, with as much as 98% of soybean meal (the residue left after oil extraction) going into livestock feed [4]. When it comes to predicting overall feed quality, crude protein alone was found to be less important than a balance of essential amino acids, with the U.S. Soybean Export Council identifying lysine, cysteine, methionine, threonine, and tryptophan as being the five most critical amino acids for determining feed quality [5]. Commodity soybeans traditionally contain high levels of polyunsaturated acids (~63%), which could lead to lipid oxidation and degradation during frying and storage [6]. Thus, many genetic breeding efforts are targeted at silencing the production of polyunsaturated fatty acids while increasing the production of oleic acid [6,7,8].

The ability of soybean to provide consistently high-quality protein and oil content, essential amino acids, and high polyunsaturated fatty acid composition makes it a target for substantial breeding and genome editing programs. While technological advances in high-throughput genotyping over the past decade, such as gene-editing technologies, have ushered in a new era of plant genomics, plant breeders have identified the lack of high-volume screening as a major bottleneck in phenotyping [9]. In addition to plant breeders, soybean farmers also need faster methods to phenotype soybeans. Traditionally, the price that a soybean farmer receives for their soybeans is a flat fare by the bushel and not adjusted for oil content, protein content, or amino acid profile [10]. Premium pricing can be obtained for soybeans that have a more favorable composition, including higher protein and oil content, total metabolizable energy, digestibility, batch-to-batch consistency, and amino acid profile. As a result, breeders and growers are continually developing varieties that can satisfy the market’s needs [11].

Based on these market trends, a rapid and non-destructive technology that can measure the crude protein and fat content, profile the critical amino acids and major fatty acid composition of soybeans, and assess the batch-to-batch consistency of the harvest will be valuable for both soybean breeders and farmers. Near-infrared (NIR) spectroscopy has been in use since the 1960s for analyses of compositional traits such as moisture, protein, starch, and fat content in various food groups ranging from grains to legumes to dairy products [12,13,14,15,16,17,18,19]. In soybeans, total protein and fats, some amino acids, and fatty acid profiles have been successfully measured using NIR spectroscopy [20,21,22], although performance suffered for amino acids such as tryptophan and sulfurous amino acids such as methionine and cysteine, all three of which are considered either essential or semi-essential amino acids for livestock [23]. A previous study on amino and fatty acid predictions with NIR spectroscopy showed that direct measurement of whole intact soybean seeds tends to see a lower coefficient of determinations (R^2^ = 0.06–0.83) as compared to ground soybeans (R^2^ = 0.38–0.85) [24]. Better prediction performance of ground samples as compared to whole kernel/kernels has been observed in other crops such as maize and rapeseed [25,26,27,28]. A major challenge with intact seeds was the infrared scattering effects that arise from uneven surfaces of whole seeds and kernels.

Previous studies of NIR spectroscopy methods for soybean characterization employed benchtop spectrometers, which constrains analysis to the laboratory instead of the field. Advancements in microchip technology have propelled a variety of compact NIR devices into the market, where a small handheld device can contain not just the light source, interferometer, and photodetector, but also embedded electronics for system control and data processing. These portable optical systems offer spectral resolutions that often parallel their benchtop counterparts while providing similar or superior performance [29]. Successful prediction models built using these compact sensors could open the door to on-the-go soybean protein and fat quality monitors, such as systems mounted on a combine harvester. Not only can this increase the efficiency of breeding and cultivar selection, but it also provides a quick way to measure protein and oil quality at point-of-sale, thereby evolving the sale of soybeans toward a value-pricing model. Detailed information on the application of the portable and handheld NIR spectrometers can be found in the literature [30,31,32].

The objective of this study was to investigate the feasibility of using real-time, field-deployable, handheld NIR spectrometers to develop partial least squares regression (PLSR) models to rapidly quantify crude protein, essential amino acids (lysine, threonine, methionine, tryptophan, and cysteine), total fat content, major fatty acid composition, and moisture content in ground soybeans and to identify high-oleic soybeans from their conventional counterparts.

## 2. Materials and Methods

### 2.1. Sample Preparation

A variety of commercial and regionally sourced soybean samples were acquired for this study. Conventional (*n* = 30) and genetically modified Plenish soybeans (high-oleic variety) (*n* = 30) were kindly provided by DuPont Pioneer’s Plenish Division. To increase the protein variability, the Ohio Soybean Council (OSC) supplied additional soybeans (*n* = 47) representative of the Midwest region. Furthermore, we included other soy products (*n* = 15), including soy isolates, soy concentrates, and soy supplement drink powders that were purchased from online vendors. The soybean samples were blended with liquid nitrogen and homogenized using a Waring Lextra 2 speed blender (East Windsor, NJ, USA) to produce a fine powder. Samples were blended for 60 s on high speed, with manual stirring at every 20-s interval. All the samples, including blended soybeans and powdered soy products, were sieved through a screen (US mesh size #30, or 0.595 mm) to maintain uniform particle size, and those samples were used both for spectroscopic and chemical reference analysis.

Due to the limited resources, each type of reference analysis could only be implemented to a selective number of samples (individual amino acids = 32; total protein = 92; individual fatty acids = 96; fat = 60; and moisture = 60).

### 2.2. Reference Analysis

Ground samples were sent to the Service Testing and Research (STAR) Laboratory at The Ohio State University, where the Dumas combustion method (AOAC 992.23) was performed for the quantitative determination of nitrogen. The output was multiplied by a nitrogen conversion factor of 5.71 as recommended by the USDA, the Food and Agriculture Organization (FAO), and World Health Organization (WHO) to obtain crude protein content [33,34,35].

Amino acid characterization was conducted by the Agricultural Experimental Station Chemical Laboratories at the University of Missouri, Columbia, using the AOAC Official Method 982.30. Lysine and threonine were determined using the standard method of acid hydrolysis with 6 M HCl and followed by hydrolysis for 24 h at 110 °C, with the hydrolysate dried under vacuum and dissolved in buffer. For methionine and cysteine analysis, performic acid was used instead of HCl for hydrolysis. For tryptophan analysis, alkaline hydrolysis was performed using 4.2 N NaOH. All resulting hydrolysate underwent cation-exchange chromatography coupled with post-column ninhydrin derivatization and quantitation. The amount of each amino acid was calculated and expressed as mg of amino acid/100 mg of soybean sample, equivalent to a percentage-by-wet-mass basis.

To determine the total fat content, about 10 g of ground soybean was placed in a 33 mm × 80 mm single-thickness cellulose extraction thimble (Whatman, Buckinghamshire, UK). The thimble was placed in a Soxhlet extraction tube with 125 mL of petroleum ether. Extraction was performed for 6 h per AOAC Method 945.16. Total fat content was calculated as the weight of the initial minus final weight of the thimble with the sample divided by the initial sample weight multiplied by 100. The petroleum ether and fat mixture were then placed on a rotary evaporator to remove the solvent, and the fat was pipetted from the bottom of the flask. The fat was stored at 4 °C until needed for further gas chromatography (GC) analysis.

The determination of fatty acid profile in extracted fats was accomplished with a fatty acid methyl ester (FAME) derivatization. For this purpose, approximately 100 µL fat sample diluted in 10 mL of hexane and 100 mL 2 N potassium hydroxide in methanol was vortexed for 30 s. A 1.5 mL aliquot was transferred to a 2 mL micro-centrifuge tube, and a pinch of sodium sulfate anhydrous was added to the mixture. The centrifuge tube rotated at 13.2 rpm for 5 min at room temperature using an Eppendorf 5415 R Centrifuge (Eppendorf North America, Hauppauge, NY, USA). A 1 mL aliquot of the hexane portion was collected in a 1.5 mL amber colored glass GC vial with Teflon screw-top cap. The major fatty acids were quantified using an Agilent HP-6890 series (Santa Clara, CA) gas chromatograph (GC) equipped with a flame ionization detector (FID), an HP-G1513A autosampler and a sample tray. The fatty acids were separated through an HP-88 60 m × 0.25 mm × 0.2 mm column (Agilent 112–8867) using helium as the carrier gas. The injection volume was 1 mL, with a split ratio of 20:1. The oven conditions were 110 °C for 1 min following by increase to 220 °C (5 °C min^–1^) and held at that temperature for 15 min. The injector and the detector temperature were set at 220 °C and 250 °C, respectively. The identification of the fatty acids was carried out by comparing the retention times of each peak against reference standards (Supelco 37 Component FAME Mix, Sigma Aldrich, St. Louis, MO, USA). All chemicals and solvents used in this study were purchased from Fisher Scientific (Waltham, MA, USA). The concentration of each fatty acid was calculated based on the percentage area under the peak.

The moisture content of the ground soybean samples was determined using a Metrohm, 915 KF Ti-Touch Karl-Fischer (Herisau, Switzerland) automatic titrator, following the AOAC Official Method 2001.12, and expressed as a percentage.

### 2.3. NIR Spectroscopic Analysis

**Figure 1:**
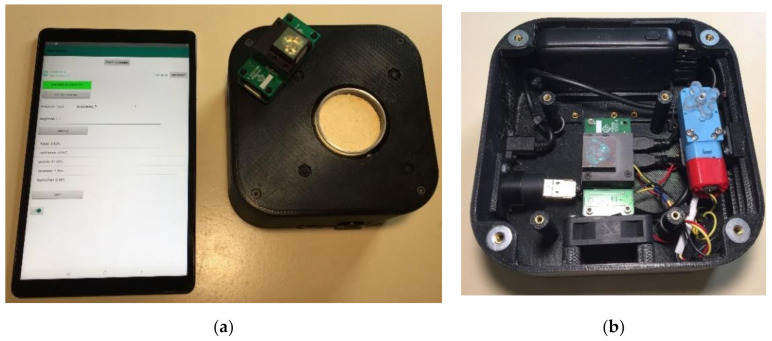
Photograph of the handheld NIR prototype sensor used for soybean samples spectra collection (**a**) and an inner view of the spectrometer housing (**b**).

NIR spectral data were acquired using a handheld prototype instrument (Figure 1a,b) based on the NeoSpectra Micro (Si-Ware Systems, Cairo, Egypt) spectrometer. This compact Fourier Transform Near-Infrared (FT-NIR) spectrometer comprises a single-chip Michelson interferometer with monolithic opto-electro-mechanical structure and a single uncooled indium-gallium-arsenide (InGaAs) photodetector. The prototype instrument also includes a sample rotation stage to enable spatial averaging over a variety of view angles, mitigating effects of spatial heterogeneity. Additional components include a motor, a USB port, a cooling fan, and a battery pack that enables 12 h of operation without charging. The prototype unit uses Bluetooth wireless data transfer to an Android tablet. Diffuse reflectance spectral measurements were accumulated over a spectral range of 1350–2560 nm with spectral resolution of 16 nm. A variety of integration times were tested, and the best reproducibility and signal-to-noise ratio was observed at 20 s. For each sample, spectra were collected in triplicate.

**Figure A1:**
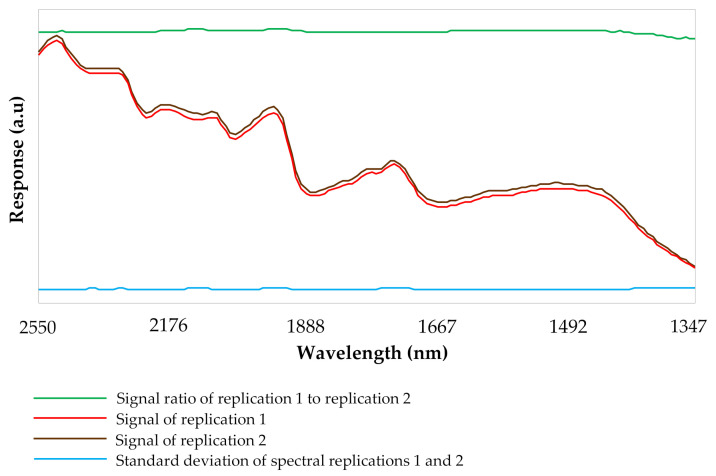
Noise level information by the signal ratio and taking the standard deviation of a random sample’s spectral replications and their corresponding spectra.

**Figure A2:**
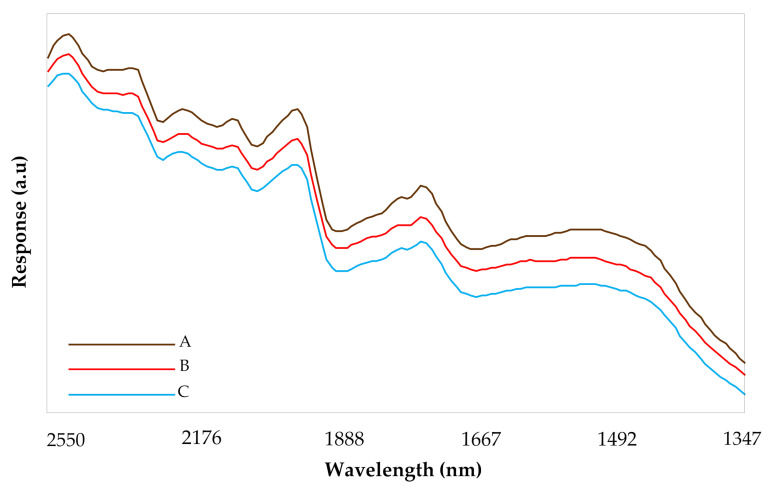
The reproducibility of the spectral measurements for a random sample that is tested with 3-h intervals. Spectra were collected at given times; A: 11 p.m. B: 2 p.m. C: 5 p.m.

A Duroplan (DWK Life Sciences, Mainz, Germany) 60-mm diameter Petri dish was used to hold the samples during NIR measurements. We found that it was important to use distortion-free glassware for sample presentation, as some Petri dishes have ring-shaped distortions on the bottom, which can cause scattering and affect measurement accuracy. Ground soybean was added to the Petri dish until ¾ full and gently tapped on the bench ten times or until the sample is compact and no visible cracks can be seen from the bottom of the Petri dish. The sample thickness was maintained at 1 cm. The Petri dish was placed on a rotating plate, which slowly rotates the dish during the spectral collection, with a 1 mm gap between the Petri dish and the spectrometer’s optical window. The background spectra were collected using a highly Lambertian diffuse reflectance standard (99% reflectance value, Spectralon, Labsphere, North Sutton, NH, USA) to eliminate the environmental changes. Noise levels of the collected spectra were observed by rationing and taking the standard deviation of two replications of a random sample and the visual representation is given in Appendix A, Figure A1. The reproducibility of the spectral measurements was evaluated by collecting spectra with 3 h intervals (Appendix B, Figure A2).

### 2.4. Statistical Analysis

The effect of gene editing on soybean crude protein content, total fat, and major fatty acid composition was evaluated by using independent samples t-test to determine whether there were statistically significant differences between groups (high oleic, GMO vs. non-GMO). If the probability values (*p*-value) were lower than 0.05, the groups were considered to be significantly different. Statistical analyses were performed using IBM SPSS Statistics software version 26.0 (IBM Co., Armonk, NY, USA).

### 2.5. Multivariate Analysis

The NIR spectral data were collected in the GRAMS (.spc) file format and analyzed using a commercial multivariate statistical analysis software package (Pirouette version 4.5, Infometrix Inc., Bothell, WA, USA). Quantification models of total crude protein, essential amino acids, total fat, major fatty acid composition, and moisture content were generated using PLSR. Full cross-validation (leave-one-out approach) was used to validate the calibration models internally. Soybean samples were randomly divided into calibration (80% of the total samples) and independent validation (the remaining 20% of the total samples) sets to determine the robustness of the generated models. The two replications of the same sample were used either in the calibration set or in the external validation set. An NIR spectrum consists of thousands of data points, and PLSR extracts a set of dependent variables (latent variables or factors) from that spectrum, providing an information rich-data set by reducing the dimensionality and solving the problem with high-collinearity [36]. The performance of the generated prediction models was evaluated by root mean square error of cross-validation (RMSECV), root mean square error of prediction (RMSEP), loading vectors, the correlation coefficient of cross-validation (R_CV_), outlier diagnostics, leverage, and residual analysis. For each model, the optimal number of factors was defined as the number that results in the first local minimum RMSECV. An ideal prediction model should have small values of RMSECV as well as R_CV_ close to unity [16]. Samples with abnormal standard residual patterns and high leverage were re-analyzed and excluded from the model if necessary; therefore, the number of samples in each model could be different.

The classification analysis between genetically modified (high-oleic) and conventional soybeans used soft independent modeling of class analogy (SIMCA). SIMCA is a supervised classification algorithm using principal component analysis (PCA) to cluster groups based on their spectral characteristics. SIMCA uses prior knowledge of class memberships and applies that information to assign new samples to the group with the lowest residual variance. The discriminating power plot identifies which variables (spectral bands) have a widespread impact on the classification of the groups of genetically modified (high-oleic) and conventional samples. The performance of the generated predictive models was evaluated based on their 3D class projection, interclass distance, misclassifications, and residuals. Tight and well-separated clusters with an interclass distance >3 indicated that the classes are significantly different from each other [37]. High-oleic and conventional samples were randomly divided into calibration (80%) and validation (20%) sets. The accuracy and robustness of the generated calibration model were evaluated using the external validation set. Furthermore, the classification model performance was also evaluated by calculating the specificity and the sensitivity of the results from the validation model. Sensitivity is the ability of the model to identify its samples (true positives), and specificity is the ability of the model to distinguish external samples (true negatives) [38].

All spectral data for the PLSR and SIMCA modeling were mean-centered by calculating the average of all points within a spectrum and subtracting that average value from each point to remove unnecessary information and enhance sample-to-sample differences [39]. For all the PLSR models (total crude protein, crucial amino acids, fat, fatty acids, and moisture content), we found a combination of normalization (2-norm × 100) and 2nd derivative (Savitzky–Golay polynomial filter with a 21-point window) transformations was most effective for data pretreatment. Taking the normalization and 2nd derivative transformation can resolve overlapping peaks by enhancing subtle peak shoulders that can highlight sample-to-sample differences and eliminate the baseline shift [40]. The data for the SIMCA modeling were only transformed with normalization (2-norm × 100) after the mean-centering.

## 3. Results

### 3.1. Characterization of Soybean Samples

Table 1 summarizes the reference analysis results for levels of essential amino acids (lysine, threonine, methionine, tryptophan, and cysteine), total protein, major fatty acids (palmitic, stearic, oleic, linoleic, and linolenic), fat, and moisture for all samples, including soybean, soy isolate, soy concentrate, and soy supplement drink powders.

Although the soybeans used in this research were sourced from various cultivars and growing regions across the Midwest region, they still exhibited a narrow range of protein and essential amino acid levels. This prompted us to include other powdered forms of soy products, including isolate, concentrate, and soy supplement drink powders into our analysis to extend the range of values to build a more accurate predictive model. The soybean protein content ranged from 32.48–37.40%, with an average of 34.12 ± 0.89% (Table 1). The protein contents for the soybeans were within the range reported by the USDA (32.00–38.50% with an average of 34.11 ± 0.67%) and Banaszkiewicz (2011) (32–43.6%) [41,42]. On the other hand, some other researchers reported slightly higher protein content; Singh and others (2008) mentioned that the average protein concentration in the soybean is 38% while Preece and others (2017) stated the average is 40% [43,44]. Temperature, solar radiation, water availability, soil nutrient supply, and genotype are the main factors that affect the protein and the amino composition of the soybeans, which may explain the differences in the protein content on different studies [45,46]. The other possibility of having a lower protein content from some of the studies could be using a different nitrogen-protein conversion factor. Although USDA and FAO suggest a soybean nitrogen-protein conversion factor of 5.71, as used in this study, some studies in the literature use 6.25 to determine the protein concentration and therefore reported higher protein concentrations, such as 38.5–40.8% [47], 37.0–43.6% [48], and 36.8–39.0% [49].

The essential amino acid (lysine, threonine, methionine, tryptophan, and cysteine) content of soybean samples and soy products are reported in Table 1 and exhibited a narrow range. A previous study that compared 14 different soybean cultivars also found a narrow range of values for threonine, cysteine, methionine, lysine, and tryptophan. Additionally, the study found no significant difference in cysteine, methionine, lysine, and tryptophan between public sector cultivars and other cultivars, except for threonine (*p* < 0.001) [50]. This explains why although various cultivars of soybeans were sourced for our investigation, sample-to-sample variability in all five amino acids was small. A separate study showed that climatic variables during soybean growth were more impactful on amino acid composition, with the cysteine levels ranging from 0.14 to 0.68%, threonine ranging from 0.87 to 2.19%, methionine from 0.31 to 0.85%, tryptophan from 0.30 to 0.80, and lysine from 0.88 to 3.92% [51]. All these ranges are much wider than exhibited by our soybean samples, thus it is recommended to source soybeans with a diverse set of amino acid profiles by capturing soybeans grown in varied climatic conditions, not just varying cultivars. The soybean samples’ fat content ranged from 16.07 to 16.97%, with an average of 16.35 ± 0.18 (Table 1), which is consistent with ranges reported by other researchers [42,48,52,53]. The major fatty acid composition of the soybeans (Table 1) was similar to values reported in previous studies [54,55,56]. The moisture content of ground soybean samples ranged from 5.30 to 5.68%, with an average of 5.49 ± 0.09% (Table 1). The optimum harvest moisture content of the soybean is 13%, but soybeans consistently lose their moisture content during storage, explaining the low moisture content [57].

The high-oleic and the conventional soybean varieties tested in this study had an average of 34.2 ± 0.6% and 33.7 ± 0.6% protein content, respectively, which indicated a significant difference (*p* < 0.05) (Table 2). High-oleic varieties exhibited higher protein content than the conventional samples, indicating that the genetic modification of the soybean seeds that increased levels of oleic acid also increased protein levels significantly. Similarly, La and others (2014) found increased protein levels after genetic modification of soybeans [58]. Like protein content, the fat content in the high-oleic varieties exhibited greater increase than the conventional varieties (Table 2). La and others (2014) also reported elevated fat content in high-oleic varieties [58]. The high-oleic soybeans showed a significant difference (*p* < 0.05) in palmitic, stearic, oleic, linoleic, and linolenic acids compared to conventional soybeans. The fatty acid composition of the high-oleic and conventional soybeans determined in this study was similar to the literature [54,59]. In high-oleic soybean varieties, the oxidative stability was improved with an average of 53% increase in oleic acid as opposed to an average 49% decrease in polyunsaturated fatty acids (linoleic and linolenic acids) levels compared to their conventional counterpart (Table 2).

### 3.2. Characterization of NIR Spectra

**Figure 2:**
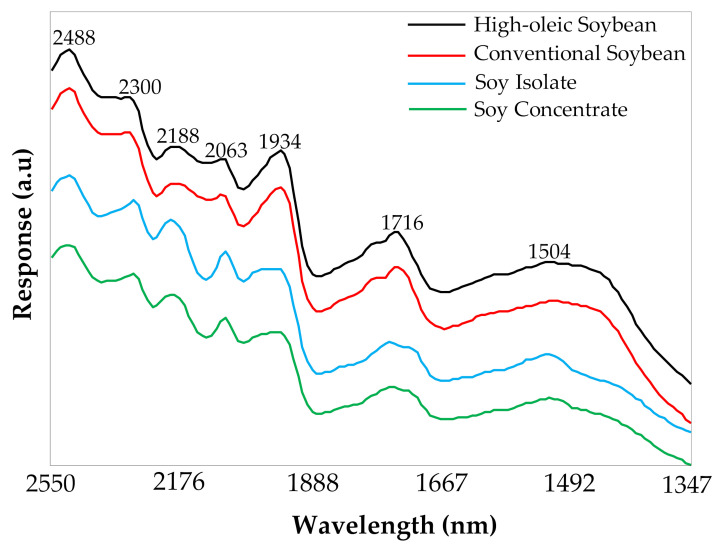
Raw NIR spectra of ground soybeans (high-oleic and conventional varieties), soy isolate, and soy concentrate.

Figure 2 shows example NIR spectra collected from ground soybeans (high-oleic and conventional varieties), soy isolate, and soy concentrate. The absorptions in the NIR region correspond to vibrational transitions between the fundamental energy level and energy levels of overtone and combination bands [60]. The region between 1695 and 1786 nm is associated with the first overtone of C–H groups of fatty acids [61]. The region between 1923 and 1961 nm is associated with the O–H functional group (1st overtone of the combination mode), and 1786–2000 nm corresponded to the C–H functional group (1st overtone of CH_3_ and –CH=CH–, of fatty acids) [16]. The 2000–2222 nm range includes the stretching and combination vibrations of N–H and C=O bonds corresponding to proteins that are assignable to a combination of amide I and amide II bands. The major band at 2063 nm corresponds to N-H bending and stretching combination bands [16,29,62]. The region between 2273 and 2480 nm is associated with combination bands of C–H groups, typically from fatty acids and carbohydrates [16,29,61].

Spectral differences associated with the protein and fat content of soy isolates, concentrates, and ground soybean samples were mainly located at 2040–2220 nm, 1890–2000 nm, and 1790–1695 nm (Figure 2). The soy isolates and concentrates had protein levels of ~90% and 70%, respectively, while the soybean samples had ~34% protein. On the other hand, the fat content in soybeans (~16%) was also higher than soy isolates and concentrates (~3%) [63,64]. The bands in the 2040–2220 nm region, specifically the bands at 2188 nm and 2063 nm were notably higher for the soy isolate samples, which had the highest amount of protein. Our visual observations were consistent with to the band assignments of 2188 nm, which is related to the N−−H bend second overtone, C−−H stretch/C==O stretch combination, and C==O stretch/amide III combination of the protein structures and 2063 nm, which is associated with the N−−H bend second overtone or N−−H bend/N−−H stretch combination of the protein structures [65]. The bands in the 1890–2000 nm and 1790–1695 nm regions were lower for the isolate and the concentrate because of their lower amounts of fat content. In the literature, those regions are assigned to the fatty acid structures [16]. Even though there was a significant difference in the fat and protein content between the high-oleic and conventional soybean samples, no marked visual difference was observed on the raw spectral profiles (Figure 2).

### 3.3. SIMCA Classification Model for High-Oleic vs. Conventional Soybeans

**Figure 3:**
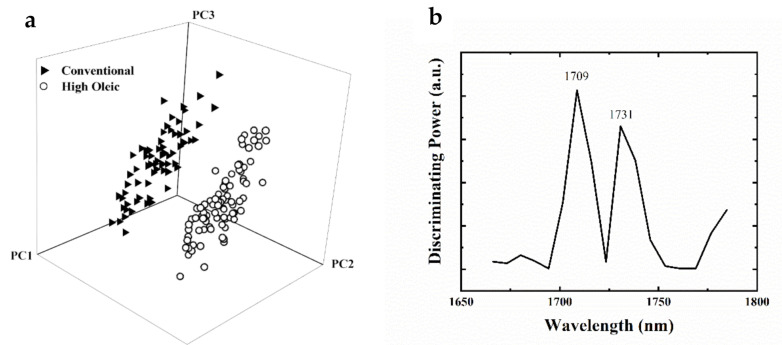
Soft independent modeling of class analogy (SIMCA) 3D projection plot for high-oleic and conventional soybean varieties (**a**) SIMCA discriminating plot based on the NIR spectra of high-oleic and conventional soybean samples using the handheld NIR sensor, showing bands and regions responsible for class separation (**b**).

To discriminate the genetically modified (high-oleic) samples from the conventional soybean samples, NIR spectral data were evaluated using soft independent class analogy (SIMCA) based on information obtained by GC analysis. Figure 3a shows a SIMCA projection plot for the NIR results using the first three principal components (PCs), indicating well-separated classes using the first three factors determined by the cross-validation leave-one-out approach. The projection shows compact clusters for high oleic and conventional soybean samples, giving an interclass distance (ICD) of 10.1. The ICD is a unitless value that indicates how well the groups are separated from each other in the multivariate space. An ICD greater than 3.0 suggests that the two tested groups are significantly different and thus can be assigned to separate classes [37]. In brief, high-oleic and conventional soybean groups were largely independent of each other, requiring only three principal components (PCs) to explain 98% of the variance within groups. The cross-validated SIMCA model indicated zero misclassifications and zero no-match samples. The discriminating power graph in Figure 3b demonstrates the variables (wavelengths) that can be characteristic of the specific chemical components responsible for the separation between the high-oleic and conventional soybean groups [66]. The discriminating power plot (Figure 3b) showed that the classification of high-oleic and conventional soybean samples is explained by the bands centered at 1731 and 1709 nm, which corresponds to the C–H stretching vibrations of lipids (1st overtone of fatty acids) [65,67].

The classification model’s prediction performance was evaluated using an external validation set that comprises 20% of the whole samples in each category (6 high-oleic and 6 conventional samples). The external validation showed that the SIMCA classification model had no misclassification, no unmatched or unmodeled samples, and 100% accuracy for predicting the new samples. Furthermore, the SIMCA model’s predictive performance showed 100% specificity and sensitivity in distinguishing the high-oleic samples or conventional samples in the correct classes.

### 3.4. Regression Models

PLSR models were generated using the spectra collected by the handheld NIR instrument and the reference analysis results for each quality parameter, including crude protein, fat, and moisture contents; essential amino acids; and major fatty acids profile. Samples were randomly divided into two groups as calibration (80% of the total sample size) and external validation (the remaining 20%) before the PLSR modeling. The calibration model was cross-validated (leave-one-out), and the external validation set used to evaluate the robustness of the generated calibration models. During the PLSR model development, it was critical to select the relevant spectral range and the optimum number of factors. The relevant spectral range selection improves the quality of predictions by removing the highly collinear neighboring wavelengths [68]. Therefore, the region used to generate each model was related explicitly to the investigated components. The optimum number of factors for each model was also chosen specifically to eliminate under or overfitting. Table 3 shows the performance statistics of the PLSR calibration and external validation models that were obtained for each relevant constituent of soybeans, soybean isolates, concentrates, and soy supplement drink powders. As mentioned previously, due to the limited resources, every reference test could not be applied to each sample. The number of samples employed in each reference test was as follows; individual amino acid analysis (*n* = 32), total protein analysis (*n* = 92), individual fatty acids (*n* = 96), fat (*n* = 60), moisture (*n* = 60).

**Figure 4:**
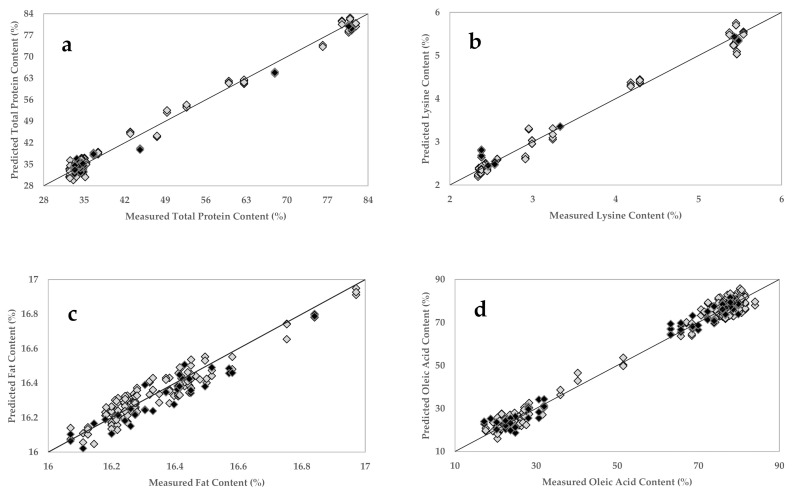
Partial least squares regression (PLSR) calibration and external validation plots for total protein (**a**), lysine (**b**), fat (**c**), and oleic acid (**d**) contents in soybean samples using the handheld NIR sensor. Grey diamonds represent samples in the calibration set; black diamonds represent samples in external validation set.

**Figure A3:**
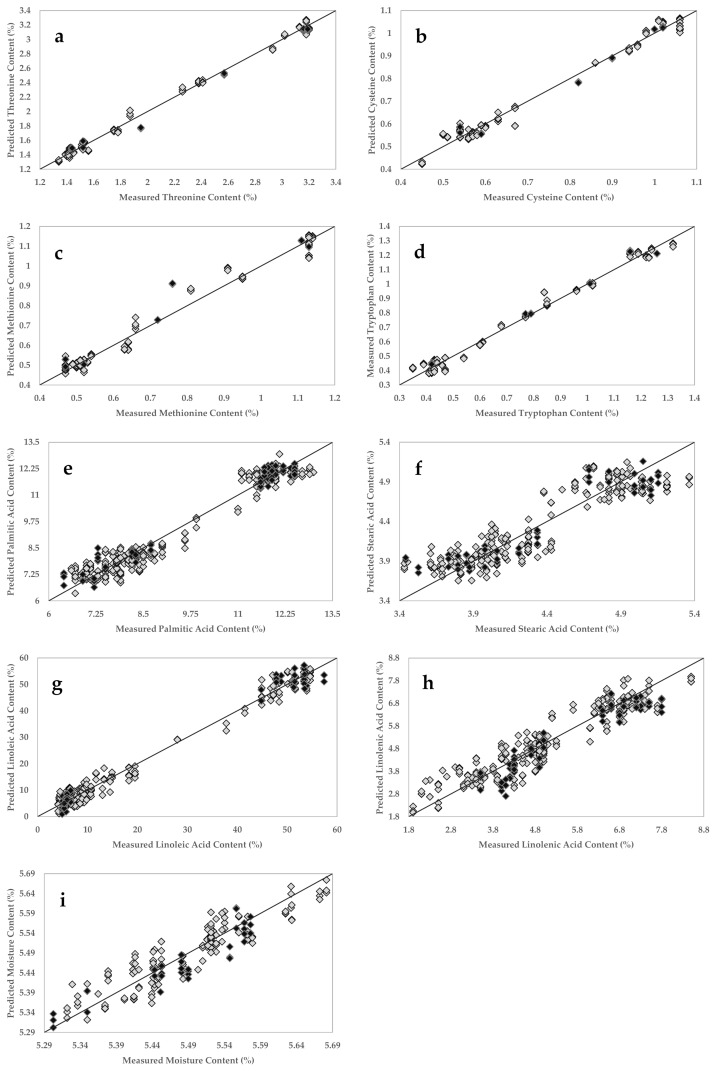
Partial least squares regression (PLSR) calibration and external validation plots for threonine (**a**), cysteine (**b**), methionine (**c**), tryptophan (**d**), palmitic acid (**e**), stearic acid (**f**), linoleic acid (**g**), linolenic acid (**h**), and moisture (**i**) contents in soybean samples using the handheld NIR sensor. Grey diamonds represent samples in calibration set; black diamonds represent samples in external validation set.

A full cross-validated total protein calibration model was generated using the dataset within a relevant spectral range (2294–1876 nm) and employing four factors (Table 3) (which explains 97.34% of the variation) as an optimal number of principal components (PCs). Figure 4a shows the PLSR plot developed for the total protein content, with a correlation coefficient (R^2^) of 0.99 and RMSECV of 1.51%. Ingle and others (2016) reported the use of a NIR probe analyzer combined with PLSR to generate predictive models for the determination of protein content (range 22–90%) of 17 protein powder mix products with SECV of 3% and R^2^ of 0.99 [69]. Benchtop FT-NIR units’ prediction of protein content exhibited R^2^ of 0.94 and SECV of 0.26% [48], and R^2^ of 0.81 and root mean square error of the calibration (RMSEC) of 0.81% [70]. The calibration models for the essential amino acids (lysine, threonine, methionine, tryptophan, and cysteine) were generated using the spectral range between 2307 and 1978 nm, and four to six factors (Table 3) were used to explain 99.66–99.96% of the variance. Figure 4b shows the PLSR plot developed for the lysine amino acid content and providing an R^2^ of 0.99 and an RMSECV of 0.17% (Table 3). Furthermore, the other amino acids exhibit R^2^ of 0.99–1.00 and RMSECV of 0.05, 0.04, 0.04, and 0.03%, respectively (Table 3). The PLSR plots for threonine, cysteine, methionine, and tryptophan amino acids are given in Appendix C, Figure A3. A previous study on amino acid prediction with NIR spectroscopy showed that the direct measurement of whole intact soybean seeds tends to produce reduced coefficients of determination (R^2^ = 0.06–0.83) compared to ground soybeans (R^2^ = 0.40–0.85) [24]. In another study, a benchtop NIR spectrometer was used to predict the crude protein and amino acid contents in the ground soybean samples and produced R^2^ of 0.84–0.98 and standard error of cross-validation (SECV) of 0.015–0.092% for the tested amino acids and R^2^ of 0.99 and SECV of 0.545 for crude protein [26]. Balastreri and others (2016) determined total protein, lysine, and methionine amounts in ground soybeans using a benchtop unit and obtained 0.995, 0.975, and 0.943 for R^2^ and 0.172, 0.012, and 0.04% for SECV, respectively [71].

Total fat content model was generated by using the spectral range from 1859 to 1680 nm and six factors (Table 3), which explain 99.97% of the variance. The PLSR plot is shown in Figure 4c and provides R^2^ of 0.95 and RMSECV of 0.05% (Table 3). We obtained better model performances than previously reported studies with benchtop units [16,70,72]. To generate the fatty acid composition models, the spectral range (1859–1680 nm), which contains signatures related to fatty acids, was selected. The models used five to six factors (Table 3) to explain 99.64–99.89% of the variance. Figure 4d shows the PLSR plot developed for the oleic acid, providing R^2^ of 0.99 and RMSECV of 3.04%. The other major fatty acids, including palmitic, stearic, linoleic, and linolenic acids provided R^2^ of 0.91–0.99 and RMSECV of 0.21–2.48% (Table 3) and the PLSR plots of these fatty acids are given in Appendix C, Figure A3. The performance of the calibration models that were developed in this study using the handheld NIR sensor with ground soybean samples is superior to previously reported studies conducted on ground [24], whole [73], and soybean cotyledons [74] using benchtop NIR equipment.

The moisture content model was generated using spectra between 1825 and 1440 nm and six factors. The model was able to explain 98.79% of the variance and provided R^2^ of 0.91 and RMSECV of 0.04 (Table 3) and the PLSR plot for the moisture models is given in Appendix C, Figure A3. Our study showed superior performance in the determination of moisture content than Ferreira and others (2013) [70].

Generated calibration models were externally validated using an independent set of samples, and their robustness was evaluated. For each trait, similar model performances, including R_cv_, R_Pre_, SECV, and SEV, both for the cross-validated calibration set and externally validated were obtained (Table 3). Figure 4 and Appendix C, Figure A3 show how the external validation set samples distribution within the range of the calibration set. Additionally, the predictive performances and the robustness of the generated models were further evaluated through residual predictive deviation (RPD) and range error ratio (RER) [75]. The RPD is the ratio between the standard deviation of the reference data in the calibration set and the RMSEP, and the RER is the range of the reference data in the validation set to the RMSEP [76]. In general, higher RPD and RER indicate a more accurate and robust model [76,77]. Values for RPD between 2.5 and 4.9 are acceptable for screening purposes, 5 to 6.4 indicates good prediction for quality control applications, and the values above 6.5 are suitable for process control applications [77]. On the other hand, values greater than 4 for RER are acceptable for sample screening, more than 10 is suitable for quality control, and more than 15 is for quantification purposes [78]. Accordingly, the proposed NIR spectrometer is a suitable tool for quantification and process control applications for all essential amino acids (except methionine), total protein, oleic acid, and linoleic acid contents (Table 3). Methionine, stearic acid, linolenic acid, and moisture content models can be used for rough screening purposes (Table 3). According to the RER values, palmitic acid and fat contents can be predicted with reasonable accuracy, and the models can be used for quality control applications, but their RPD levels indicate lower accuracy (Table 3), which could be related to the random sample selection in the validation set (low standard deviation).

## 4. Conclusions

Our study supports the use of a novel portable NIR spectrometer based on a monolithic opto-electro-mechanical structure and InGaAs photodetector for assessing protein quality including crude protein, essential amino acids (threonine, cysteine, methionine, lysine, and tryptophan), fat, moisture, and fatty acid (palmitic, stearic, oleic, linoleic, and linolenic acids) in commercial soybeans and soy products. Combining reference test results with NIR spectra along with multivariate analysis, we successfully developed prediction models with strong correlation between reference tests and predicted values (R_Pre_ ≥ 0.92). All prediction models exhibited high precision with low RMSEP values (0.02–3.07%), although widening the range of crude protein and amino acid levels would certainly make the model more reliable. Furthermore, our instrument can discriminate between high-oleic and conventional soybean samples without misclassification. The performance of the PLSR models developed by the handheld instrument was shown to be equivalent and, in some instances, superior to models developed from benchtop infrared systems in other studies. A handheld spectrometer can provide soybean breeders and growers with a tool that can be taken to the field for real-time analysis of soybeans to facilitate faster decision-making.

## Figures and Tables

**Table 1:** Reference analysis results for essential amino acid (lysine, threonine, methionine, tryptophan, and cysteine), total protein, major fatty acid (palmitic, stearic, oleic, linoleic, and linolenic), fat, and moisture content in soybeans and soy products.

Parameter (%) *		Minimum	Maximum	Mean	STDEV **	CV% ***
Threonine	Soybean	1.34	1.56	1.45	0.06	4.17
	Soy Products	1.75	3.20	2.64	0.54	20.46
Cysteine	Soybean	0.45	0.60	0.55	0.04	7.20
	Soy Products	0.63	1.06	0.92	0.14	15.52
Methionine	Soybean	0.47	0.64	0.51	0.04	5.02
	Soy Products	0.63	1.14	0.95	0.20	22.39
Lysine	Soybean	2.34	2.57	2.43	0.07	2.88
	Soy Products	2.91	5.54	4.50	1.08	24.37
Tryptophan	Soybean	0.35	0.54	0.44	0.04	10.04
	Soy Products	0.60	1.32	0.99	0.23	23.31
Total Protein	Soybean	32.48	37.4	34.12	0.89	1.81
	Soy Products	42.96	81.91	67.39	14.46	21.45
Palmitic Acid	Soybean	6.22	13.4	9.19	2.57	22.93
Stearic Acid		3.53	5.21	4.40	0.54	11.79
Oleic Acid		17.60	84.00	52.83	28.05	42.04
Linoleic Acid		4.10	57.40	27.29	22.88	91.23
Linolenic Acid		1.88	8.19	4.53	2.43	30.38
Fat		16.07	16.97	16.35	0.18	1.11
Moisture		5.30	5.68	5.49	0.09	1.59

* Individual amino acids (threonine, cysteine, methionine, lysine, and tryptophan), total protein, fat, and moisture are given in % wet basis; fatty acids (palmitic, stearic, oleic, linoleic, and linolenic) are given in % peak area from the GC analysis. ** Standard deviation. *** Coefficient of variation.

**Table 2:** Total protein, fat content, and fatty acid composition comparison of high-oleic and conventional soybean samples.

Parameter (%) *	High-Oleic	Conventional	*p*-Value **
Total Protein	34.17 ± 0.61	33.66 ± 0.60	0.000
Fat	16.42 ± 0.19	16.27 ± 0.10	0.000
Palmitic Acid	7.00 ± 0.52	11.95 ± 0.69	0.000
Stearic Acid	3.87 ± 0.33	4.91 ± 0.23	0.000
Oleic Acid	79.25 ± 2.00	23.35 ± 3.65	0.000
Linoleic Acid	5.99 ± 1.32	51.61 ± 3.13	0.000
Linolenic Acid	2.21 ± 0.32	7.09 ± 0.53	0.000

* Total protein and fat contents are given in % wet basis; fatty acids (palmitic, stearic, oleic, linoleic, and linolenic) are given in % peak area from the GC analysis. ** indicates the significant difference between high-oleic and conventional soybeans (*p* < 0.05).

**Table 3:** Statistical performance of the prediction models developed using a handheld NIR sensor for estimating various constituents of soy samples.

Parameter (%) *	Calibration Model					External Validation Model					
	Range	N ^a^	Factor	RMSECV ^b^	R_cv_^c^	Range	n ^d^	RMSEP ^e^	R_Pre_^f^	RPD ^g^	RER ^h^
Threonine	1.34–3.20	26	6	0.05	1.00	1.44–3.19	6	0.08	1.00	8.7	22.3
Cysteine	0.45–1.06	26	4	0.03	0.99	0.54–1.02	6	0.02	0.99	8.8	19.9
Methionine	0.47–1.14	26	4	0.04	0.99	0.47–1.13	6	0.07	0.97	3.8	9.7
Lysine	2.34–5.54	26	5	0.17	0.99	2.38–5.48	6	0.15	1.00	8.6	21.2
Tryptophan	0.35–1.32	26	4	0.04	0.99	0.42–1.26	6	0.04	0.99	8.2	21.2
Total Protein	32.48–81.91	73	4	1.51	0.99	33.28–81.15	18	1.64	0.99	8.3	29.2
Palmitic Acid	6.50–13.00	77	5	0.49	0.97	6.40–12.50	19	0.40	0.98	4.8	15.1
Stearic Acid	3.43–5.36	70	6	0.21	0.91	3.44–5.15	17	0.21	0.93	2.4	8.3
Oleic Acid	17.60–84.00	76	5	3.04	0.99	17.20–79.90	19	3.07	0.99	8.1	20.5
Linoleic Acid	4.10–54.60	77	5	2.48	0.99	4.90–57.40	19	2.71	0.99	7.2	19.4
Linolenic Acid	1.90–8.50	76	5	0.55	0.94	3.50–7.80	19	0.56	0.95	2.8	7.6
Fat	16.07–16.97	46	6	0.05	0.95	16.07–16.84	12	0.07	0.96	2.6	13.3
Moisture	5.32–5.68	45	6	0.04	0.91	5.30–5.58	11	0.04	0.92	2.4	7.5

^a^ Number of samples used in calibration models. ^b^ Root mean square error of cross-validation. ^c^ Correlation coefficient of cross-validation. ^d^ Number of samples used in external validation models. ^e^ Root mean square error of prediction. ^f^ Correlation coefficient of prediction for external validation. ^g^ Residual predictive deviation. ^h^ Range error ratio. * Individual amino acids (threonine, cysteine, methionine, lysine, tryptophan), total protein, fat, and moisture are given in % wet basis; fatty acids (palmitic, stearic, oleic, linoleic, linolenic) are given in % peak area from the GC analysis. RMSECV and RMSEP are in units of the predicted parameters.
